# Delayed diagnosis of glaucoma in Coffin-Siris syndrome

**DOI:** 10.1016/j.ajoc.2025.102396

**Published:** 2025-07-22

**Authors:** Angela C. Chen, Matthew Miller, Michael Kapamajian, Monica Khitri

**Affiliations:** a Division of Ophthalmology, University of California Los Angeles, Los Angeles, CA, USA; b Division of Ophthalmology, Harbor-UCLA Medical Center, Torrance, CA, USA

**Keywords:** Glaucoma, Coffin-Siris Syndrome

## Abstract

**Purpose:**

To report a case of delayed diagnosis of glaucoma in a patient with Coffin-Siris Syndrome (CSS) who did not have any other predisposing risk factors or typical anterior segment signs of congenital glaucoma.

**Observations:**

A 27-year-old male with CSS was referred to the ophthalmology department for evaluation of strabismus. Past ocular history included a diagnosis of right morning glory anomaly and bilateral hyperopic astigmatism. Initial exam in the clinic was notable for visual acuity of 20/80 in the right eye and 20/40 in the left eye. Given difficulty with exam in the outpatient setting, the patient underwent exam under anesthesia. Intraocular pressures (IOPs) were 38 mmHg OD and 23 mmHg OS at induction and gonioscopy was significant for high iris insertion and prominent iris processes in both eyes. On dilated fundus exam, the right eye had a severely pallorous and cupped optic nerve; the left eye was also cupped but less so. Elevated IOPs and nerve cupping was consistent with a diagnosis of glaucoma.

**Conclusions and importance:**

Patients with CSS may develop glaucoma and should be screened for this important ophthalmic association, even in the absence of external signs of pediatric glaucoma including an enlarged cornea, anterior segment dysgenesis, or other risk factors for glaucoma such as steroid use.

## 1 Introduction

Coffin-Siris Syndrome (CSS) is a genetic disorder characterized by aplasia or hypoplasia of the nails and terminal phalanges, developmental and growth delay, dysmorphic facial features, hypotonia, hirsutism or hypertrichosis, sparse scalp hair, and other congenital anomalies involving various organ systems. The most common family of genes implicated is *ARID*. Pathogenic variants of *ARID1B* account for approximately 37% of CSS cases. Individuals with pathogenic *ARID1B* variants typically have milder courses of CSS and normal growth whereas those with pathogenic variants in *SMARCB1* may have more severe phenotypes and growth impairment. Ophthalmologic abnormalities most commonly include ptosis, strabismus, and myopia.^1^ CSS is less commonly associated with glaucoma. Here we report a patient with CSS who was diagnosed with glaucoma at an older age given paucity of external signs suggestive of pediatric glaucoma.

## 2 Case

A 27-year-old man with a known diagnosis of CSS was referred by the Genetics department for evaluation of strabismus. He had a history of developmental delay, speech delay, and epilepsy. A panel targeting 492 genes associated with intellectual disability was performed using Next Generation Sequencing (NGS) by Fulgent Diagnostics, a CLIA-certified laboratory. This analysis identified a pathogenic heterozygous variant in the *ARID1B* gene (NM_020732.3:c.1854dup; p.Gln619Alafs∗34), which was verified by Sanger sequencing. This variant is predicted to result in loss of function of the protein product either due to protein truncation or nonsense mediated mRNA decay. While this truncating variant has not been reported and is not found in the ClinVar database, truncating variants downstream of this position have been reported to be pathogenic.^2^ The patient previously received his eye care in India, where he was diagnosed with morning glory anomaly of the right eye as well as hyperopic astigmatism in both eyes. In review of medical records, the patient underwent an exam under anesthesia 9 months prior where symmetric axial lengths (21.62 mm right eye, 21.12 mm left eye) and corneal diameters (11.0 mm both eyes) were noted. Intraocular pressures (IOPs) were 22 mmHg in the right eye and 18.6 mmHg in the left eye, albeit measurement timing after anesthesia induction was not noted and thus its accuracy could not be verified. Magnetic resonance imaging of the brain performed in India in 2015 was largely within normal limits except for a prominent retrocerebellar CSF space compatible with a mega-cisterna or arachnoid cyst.

On presentation to the authors, the patient's Snellen visual acuity was 20/80 in the right eye and 20/40 in the left eye. Both pupils were reactive to light, with a noted relative afferent pupillary defect in the right eye. Sensorimotor exam identified a moderate angle, constant V-pattern exotropia. Due to limited cooperation with the exam and inability to appropriately evaluate the patient, the decision was made to evaluate the patient under anesthesia.

**Fig. 1:**
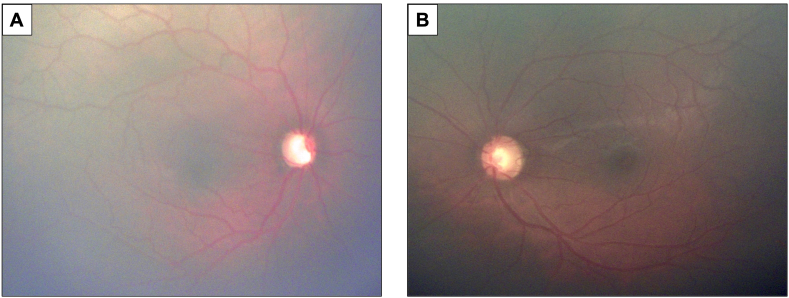
Fundus photos demonstrating A) optic nerve that is fully cupped and pallorous in the right eye and B) optic nerve that is mildly cupped and tilted in the left eye.

During exam under anesthesia, IOP was measured using a Reichert Tono-pen AVIA handheld tonometer (Depew, NY, USA) to be 38 mmHg in the right eye and 23 mmHg in the left eye just after anesthesia induction. The IOP was again measured 10 minutes after induction and was 23 mmHg in the right eye and 16 mmHg in the left eye. Central corneal thickness was 540 μm in the right eye and 551 μm in the left eye. Gonioscopy identified the iris steeply inserting just inferior to the scleral spur as well as prominent iris processes in both eyes. Cycloplegic retinoscopy revealed refractive error of -0.25 + 0.50 x180 in the right eye and +0.50 + 2.50 x170 in the left eye. Anterior segment exam was largely unremarkable. Posterior segment exam was notable for pallor and severe cupping of the right optic nerve with a cup-to-disc ratio of 0.99. The left optic nerve had a cup-to-disc ratio of 0.60 with intact rims (Fig. 1). After discussion with the patient's family regarding options for medical management versus surgical intervention, it was decided that the patient would proceed with goniotomy and start oral acetazolamide as a bridge to surgery given difficulties instilling eyedrops at home due to his level of cooperation.

## 3 Discussion

Coffin-Siris Syndrome is a rare heterogenous genetic disorder that may be associated with ocular manifestations such as strabismus, ptosis, myopia, nystagmus, cataract, microcornea, and/or buphthalmos.^3^ Very rarely has glaucoma been associated.

Dolaghan et al. reported two cases of children with *ARID1B*-related CSS who were found to have elevated IOPs and optic disc cupping.^4^ In the first case, an 8-year-old-boy with CSS developed elevated IOPs after chronic topical steroid treatment for recurrent shield ulcers from vernal keratoconjunctivitis. Despite maximal topical IOP-lowering therapy and discontinuing steroids, the child's IOPs remained elevated and only abated with Ahmed valve surgeries in both eyes. In the second case, a 4-month-old with *ARID1B*-related CSS also was found to have rising IOPs in both eyes as well as disc cupping. IOP in this case was stabilized with topical therapy alone.

In two additional reports CSS was diagnosed in patients with glaucoma and medical problems that prompted genetic screening. Diel et al. described a child with congenital anomalies and severe developmental delay whose ocular issues included secondary childhood glaucoma, ocular anterior segment dysgenesis, congenital limbal stem cell deficiency, aniridia, and cataract.^5^ He underwent whole exome sequencing and was found to have a likely pathogenic heterozygous variant in the *SOX11* gene associated with CSS. Rojananuangnit et al. described a case of bilateral secondary angle closure glaucoma and microspherophakia in a 42-year-old patient with history of intellectual disability who, for many years, was assumed to have Weill-Marchesani syndrome.^6^ Sequencing, however, showed a pathogenic variant in *ARID1B*, diagnostic of CSS.

Our patient with *ARID1B*-associated CSS presented with ophthalmic findings significant for severe primary glaucoma with elevated IOP and cupping of both optic nerves, with the right eye more affected than the left. Gonioscopy revealed a high iris insertion akin to what is seen in juvenile onset angle glaucoma, suggestive of underlying angle dysgenesis. His previous diagnosis of morning glory anomaly was likely made in error due to the excavated appearance of his nerve on presentation; however, lack of peripapillary atrophy and peripheral vessels rendered morning glory anomaly less likely. Morning glory anomaly is characterized by a funnel-shaped excavation of the optic disc with surrounding chorioretinal pigmentary changes and a central glial tuft.^7^ Retinal vessels generally maintain a normal pattern perhaps with slight displacement in optic nerve cupping whereas they frequently follow a radial pattern in morning glory anomaly.^7^ The appearance of the right optic nerve was more consistent with glaucoma, as the lamina cribrosa in younger patients is not stiff and thus more vulnerable to deep cupping in the setting of high IOPs. Based on the degree of optic nerve fiber loss, the onset of glaucoma likely began several years earlier but was undetected given the patient had difficulty cooperating for a complete exam in the outpatient setting. In contrast to the patients with CSS already described in the literature, our patient lacked external signs of pediatric glaucoma (no enlarged corneas), had no other gross signs of anterior segment dysgenesis or microspherophakia, and carried no history of steroid use or other risk factors for glaucoma. This suggests that patients with CSS may be at higher risk of glaucoma, even in the absence of other risk factors.

## 4 Conclusions

In conclusion, we report a case of severe glaucoma in a young adult patient with CSS. Given the difficulties inherent to examining patients with CSS thoroughly in the outpatient setting, ophthalmologists and clinicians caring for patients with CSS should be aware of and have a low threshold to pursue exams under anesthesia in order to screen for glaucoma earlier and prevent permanent vision compromise.

## CRediT authorship contribution statement

**Angela C. Chen:** Writing – review & editing, Writing – original draft. **Matthew Miller:** Writing – review & editing. **Michael Kapamajian:** Writing – review & editing, Supervision. **Monica Khitri:** Writing – review & editing.

## Patient consent

The patient(s)/patient's legal guardian consented to publication of the case in writing/orally.

## Disclosures

The authors report there are no competing interests to declare.

## Declaration of competing interest

The authors declare that they have no known competing financial interests or personal relationships that could have appeared to influence the work reported in this paper.
